# Gray Matter Correlates of Cognitive Performance Differ between Relapsing-Remitting and Primary-Progressive Multiple Sclerosis

**DOI:** 10.1371/journal.pone.0129380

**Published:** 2015-10-20

**Authors:** Laura E. Jonkman, Diana M. Rosenthal, Maria Pia Sormani, Laura Miles, Joseph Herbert, Robert I. Grossman, Matilde Inglese

**Affiliations:** 1 Department of Neurology, Mount Sinai School of Medicine, New York, New York, United States of America; 2 Department of Anatomy and Neurosciences, VU University Medical Center, Amsterdam, the Netherlands; 3 Department of Radiology, New York University School of Medicine, New York, New York, United States of America; 4 Department of Population Health, New York University School of Medicine, New York, New York, United States of America; 5 Department of Health Sciences, University of Genoa, Genoa, Italy; 6 Department of Neurology, New York University School of Medicine, New York, New York, United States of America; 7 Department of Radiology, Mount Sinai School of Medicine, New York, New York, United States of America; 8 Department of Neuroscience, Mount Sinai School of Medicine, New York, New York, United States of America; 9 Department of Neuroscience, Rehabilitation, Ophthalmology, Genetics, Maternal and Child Health (DINOGMI), University of Genoa, Genoa, Italy; University of Düsseldorf, GERMANY

## Abstract

Multiple Sclerosis (MS) is a chronic inflammatory/demyelinating and neurodegenerative disease of the central nervous system (CNS). Most patients experience a relapsing-remitting (RR) course, while about 15–20% of patients experience a primary progressive (PP) course. Cognitive impairment affects approximately 40–70% of all MS patients and differences in cognitive impairment between RR-MS and PP-MS have been found. We aimed to compare RR-MS and PP-MS patients in terms of cognitive performance, and to investigate the MRI correlates of cognitive impairment in the two groups using measures of brain volumes and cortical thickness. Fifty-seven patients (42 RR-MS, 15 PP-MS) and thirty-eight matched controls underwent neuropsychological (NP) testing and MRI. PP-MS patients scored lower than RR-MS patients on most of the NP tests in absence of any specific pattern. PP-MS patients showed significantly lower caudate volume. There was no significant difference in MRI correlates of cognitive impairment between the two groups except for a prevalent association with MRI measures of cortical GM injury in RR-MS patients and with MRI measures of subcortical GM injury in PP-MS patients. This suggests that although cognitive impairment results from several factors, cortical and subcortical GM injury may play a different role depending on the disease course.

## Introduction

Multiple Sclerosis (MS) is a chronic inflammatory/demyelinating and neurodegenerative disease of the central nervous system (CNS). Most patients (80–85%) experience a relapsing-remitting (RR) course with episodes of neurological dysfunction followed by complete or partial remission. About 15% to 20% of patients experience a primary progressive (PP) course characterized by a gradual worsening of neurological functions from disease onset [1,2]. Cognitive impairment affects approximately 40–70% of all MS patients [3]. While patients with RR-MS are most commonly affected by deficits of information processing speed and working memory [4,5], patients with PP-MS show not only more extensive impairment in these domains [4–7], but also in other domains such as executive functioning and verbal episodic memory, indicating a wider range of cognitive deficits [4,5]. Only a few studies have investigated the MRI characteristics of PP-MS patients in comparison to RR-MS patients. A recent MRI study performed at 7 Tesla reported no differences in lesional morphological features (count, preferred location, type, hypointense rim) between RR-MS and PP-MS [8]. However, both pathological and MRI studies have shown that cortical demylination and gray matter (GM) atrophy are more prominent in PP-MS than in RR-MS [9,10]. In addition, MRI measures of cortical lesions count, cortical thinning and gray matter atrophy are found to be associated with cognitive impairment in MS [11–16]. However, most of these studies are directed to the investigation of MRI correlates of cognitive impairment in RR-MS patients only [15,17]. Unlike RR-MS patients whose brain MRI is characterized by the predominant accrual of inflammatory/demyelinating white matter lesions [9], PP-MS are generally characterized by fewer white matter lesions, lower accrual rate of lesion and more extensive pathological involvement of gray matter and normal-appearing white matter [9,18]. Therefore, we hypothesize that cognitive impairment would have different MRI correlates in RR-MS and PP-MS patients.

The aims of our study were: i) to compare RR-MS and PP-MS patients in terms of cognitive performance; ii) to investigate the MRI correlates of cognitive impairment in the two groups using measures of lesion and brain volume, and measures of cortical thickness.

## Materials and Methods

### Subjects

Fifty-seven patients (38 women) who met the revised International Panel Criteria [19] were prospectively enrolled in the study. Forty-two patients had relapsing-remitting (RR) and fifteen had primary-progressive (PP) MS. The exclusion criteria were: i) a current or past medical or psychiatric disorder other than MS; ii) current or past substance abuse, and/or iii) MS relapse or corticosteroid use in the previous 6 weeks. An experienced neurologist who was blinded to the MRI findings assessed disability using the Expanded Disability Status Scale (EDSS [20]) within 1 week of MRI. A clinical neuropsychologist, who was also blinded to the MRI findings, administered the neuropsychological test battery detailed in the paragraph below. Thirty-three RR-MS patients and twelve PP-MS patients were under treatment with immunomodulatory agents. Of the thirty-three RR-MS patients, fourteen patients were on interferon-β1a (Avonex, Biogen Idec, Cambridge, MA USA), ten patients were on interferon-β1a (Rebif, Merck Serono, Rockland, MA, USA), seven patients were on interferon-β1a (Betaseron, Bayer HealthCare Pharmaceuticals, Montville, NJ, USA), ten patients were on glatiramer acetate (Copaxone, Teva pharmaceuticals, North Wales, PA, USA) and one patient was on natalizumab (Tysabri, Biogen Idec, Cambridge, MA USA). Of the twelve PP-MS patients, four were on interferon-β1a (Avonex, Biogen Idec, Cambridge, MA USA), two patients were on interferon-β1a (Rebif, Merck Serono, Rockland, MA, USA), four patients were on interferon-β1a (Betaseron, Bayer HealthCare Pharmaceuticals, Montville, NJ, USA) and two patients were on glatiramer acetate (Copaxone, Teva pharmaceuticals, North Wales, PA, USA). Thirty-eight frequency-matched healthy volunteers (21 women) with a similar age range and percentage of females as MS patients, were included as a control group for the MRI evaluation. Approval for this study was obtained from the Institutional Review Board of the New York University Langone Medical Center, and written informed consent was obtained from all subjects before study initiation.

### Neuropsychological testing

Cognitive functioning was assessed using a battery of neuropsychological (NP) tests, administered to all MS patients within 24 hours of MRI, examining the following cognitive domains: (I) verbal fluency, assessed with the F-A-S (FAS) test [21]; orally produce as many words as possible that begin with the letters F, A, and S in a 60 seconds timeframe (II) verbal learning and memory, assessed with the California Verbal Learning Test-II (CVLT-II) [22] which consisted of a composite score of three sub-scores: verbal learning, immediate recall and delayed recall. (III) processing speed and working memory, assessed with the Symbol Digit Modalities Test (SDMT) [23] and 3-seconds trial of the Paced Auditory Serial Addition Test (PASAT-3 secs) [24] (IV) executive functioning, assessed with the Delis—Kaplan Executive Function System [D-KEFS] Inhibition [D-KEFSI] and Inhibition Switching [D-KEFSIS] [25].

Raw NP scores were normalized using published norms [21–25] and then converted to Z-scores based on the normal distribution. For the PASAT-3 secs there were no published norms available, therefore test scores were normalized against the mean and standard deviation of 38 healthy controls, comparable in relation to age and years of schooling. Patients were defined as cognitively impaired (CI) when their Z-score on neuropsychological tests was at least 1.65 standard deviations (SD) below the norm (corresponding to the 5^th^ percentile) on two or more NP tests [26]. Otherwise, patients were categorized as cognitively preserved (CP). In addition, a composite *cognitive performance* score was calculated by adding up the *Z*-scores of all six NP tests described above to give an overall indication of cognitive performance.

Depressive symptoms were assessed with the Depression subscale of the Brief Symptom Inventory (BSI-D) [27]. The Depression subscale of the BSI assesses clinical indications of depression such as dysphoric mood and affect as well as reduced motivation and a loss of interest. T-scores of 63 or greater indicate a clinically significant level of depression that is equal to or above the 91^st^ percentile. Fatigue was assessed with the multidimensional fatigue inventory (MFI) [28], covering five dimensions of fatigue: General Fatigue, Physical Fatigue, Reduced Activity, Reduced Motivation, and Mental Fatigue. A composite score from these subscales was generated. Lower scores indicated a higher level of fatigue.

### MRI Acquisition

MRI was performed using a 3T system (MAGNETOM Trio, A Tim System, Siemens Medical Solutions, Erlangen, Germany) with an eight-channel phased-array head coil. The following sequences were collected in all subjects during a single MR session: (i) T2 dual-echo turbo spin-echo; (repetition time (TR) = 5,500 ms, echo time (TE) = 12/99 ms, 48 contiguous, 3-mm-thick axial slices with a 256 × 205 matrix, and a 220 mm × 190 mm field of view (FOV); parallel imaging acceleration factor of 2 (ii) 3D T1-weighted turbo-flash magnetization-prepared rapid-acquisition gradient-echo (MPRAGE) (TR:2400 ms, TE: 2.71 ms, TI: 900 ms; flip angle: 12°). All slice-encoding locations were acquired in a single slab, covering the whole brain, with an effective thickness of 1 mm; (iii) Post-Gd T1-weighted spin-echo (TR = 471 ms, TE = 12 ms, 50 contiguous, 3-mm-thick, axial slices with a 256 × 205 matrix, and a 220 × 220 mm2 FOV).

### Image Processing and Evaluation

#### Lesion volumes assessment

All data processing was performed off-line on a PC workstation. For all patients, T2-hyperintense and T1-hypointense lesion volume (T2-W and T1-W LV) measurements were obtained using a semi-automated segmentation technique based on user-supervised local thresholding (Jim version 3; Xinapse Systems, Northants, England, (http://www.xinapse.com)). For the T2-W LV classification, lesion borders were determined on PD-weighted images using the T2-W images as reference. T1-hypointense lesions were defined as those lesions with signal intensity between that of the GM and the CSF on T_1_-W scans [29]. In both T_2_-W and T_1_-W images, the value of total brain LV was calculated by multiplying lesion area by slice thickness.

#### Brain volume assessment

For all subjects, normalized brain volume (NBV), normalized gray, and white matter volumes (NGMV and NWMV) were measured from the MPRAGE images using Structural Image Evaluation, using Normalisation, of Atrophy (SIENAX) as described previously [30]. SIENAX automatically segments brain from non-brain matter, calculates the brain volume, and applies a normalization factor to correct for skull size [31]. To correct for misclassification of T_1_-W GM volume in presence of high T_1_-W hypointense LV [32], each T_1_-W hypointense lesion of each subject was filled with the mean intensity value of the normal appearing white matter (NAWM) present in the same slice of the lesion.

#### Cortical thickness assessment

All data processing was performed off-line on a Mac workstation. For all subjects, brain cortical thickness (CTh) and deep gray nuclei volume was measured from the MPRAGE images using Freesurfer software, version 3.0.5 (http://surfer.nmr.mgh.harvard.edu). This process consists of several stages, detailed elsewhere [33]. Briefly, the procedure included removal of non-brain tissue, Talairach segmentation of white matter, deep gray matter structures and cerebellar structures, automatic correction of topological defects and registration into an average spherical surface template. All cortical segmentations were manually checked and re-run after manual editing if errors had occurred. Thickness measurements (in mm) were obtained by calculating the closest distance between the cortical surface and the gray/white matter border. Parcellation of the brain was done using the Desikan—Killiany atlas which consists of thirty-four cortical structures per hemisphere [34]. For further analysis, we combined each area of the two hemispheres (correlation p < 0.01) and composed seven cortical areas based on a division described in Desikan et al [34]. The composed areas for analysis were the temporal lobe—medial aspect, temporal lobe—lateral aspect, frontal lobe, parietal lobe, occipital lobe, cingulate cortex and insula. Furthermore, we obtained volumetric measures (in mm^3^) from the cerebellum, thalamus, caudate, putamen, pallidum, hippocampus and amygdala.

### Statistical Analysis

Statistical analysis was performed in SPSS 20.0 (Chicago, IL). Kolmogorov-Smirnov tests and visual inspection of the histogram was used to assess normality of the variables. When the variables were normally distributed, a multivariate general linear model (GLM) was used to assess group differences. When variables were not normally distributed, the Mann-Whitney U or the Kruskal-Wallis test was used. Correlations were assessed by partial correlations controlling for age, gender and disease duration. Results were considered statistically significant at p < 0.05.

## Results

### Subject

Fifty-seven patients (38 females) with a mean age of 46.7 years (± 11.0) participated in the study. Forty-two patients had a relapsing remitting (RR) disease type with mean disease duration of 5.8 years (± 5.2). Fifteen patients had a primary progressive (PP) disease type with mean disease duration of 9.5 years (± 6.2). Thirty-seven age- and sex-matched healthy controls (mean age 49.2 ± 13.1; 21 females) were included in the study. Subjects’ demographics, clinical and radiological data are summarized in Table 1. MS patients and controls did not differ with regard to age and gender, but MS patients were significantly more impaired on the MFI (p < 0.001) and BSI (p < 0.001) scores than healthy controls. In regards to patient disease types, PP-MS patients were significantly older (p = 0.01), had longer disease duration (p = 0.023) and were more clinically disabled (p < 0.001) than RR-MS patients. RR-MS and PP-MS patients did not differ from each other on years of education, MFI (each of the five dimensions) or BSI. Fourteen MS patients (25%) were defined as CI (Z-score of -1.6 on at least 2 out of 6 tests); 9 RR-MS patients (21%) and 5 PP-MS patients (33%). Only on “verbal learning & memory” (CLVT), PP-MS patients scored significantly lower than RR-MS patients (p = 0.028), see Table 2 for a summary of scores on neuropsychological tests.

**Table 1:** Demographic and clinical characteristics of all subjects.

	Controls (n = 37)	All patients (n = 57)	RR-MS (n = 42)	PP-MS (n = 15)
**Gender (M/F)**	16 / 21	19/38	11 / 31	8 / 7
**Mean age, yrs**	49.2±13.2	46.7±11.0	43.7±9.6	54.9±10.7**
**Disease duration, yrs**	_	6.8±5.6	5.8±5.2	9.5±6.2*
**EDSS^**	_	2.5 (0.0–7.5)	1.8 (0.0–6.5)	4.0 (3.0–7.5)**
**Cognitive impaired**	_	14	9	5
**Treated/untreated**	_	45/12	33/9	12/3
**BSI**	15.4±4.0	55.9±11.3^¥^	55.5±12.1	57.1±9.2
**MFI**	81.5±15.6	68.8±15.4^¥^	69.9±16.3	65.6±12.0

Data are presented as mean ± SD, unless otherwise indicated. Abbreviations: RR-MS = relapsing-remitting MS; PP-MS = primary-progressive MS; EDSS = Expanded Disability Status Scale; BSI = Brief Symptom Inventory; MFI = multidimensional fatigue inventory.

* *p* < 0.05 when compared to RR-MS group

** *p* < 0.001 when compared to RR-MS group

^¥^ p < 0.001 when comparing to healthy controls

^^^ median and range

**Table 2:** Mean and standard deviation of Z-scores on neuropsychological tests.

	RR-MS	PP-MS
**Overall**	-1.98 ± 4.98	-3.95 ± 5.66
**FAS**	0.32 ± 1.17	0.27 ± 1.77
CLVT*	-.095 ± 1.15	-0.84 ± 0.97
**SDMT**	-0.81 ± 1.18	-1.44 ± 1.56
**DKEFS-I**	-0.15 ± 0.99	-0.10 ± 1.10
**DKEFS-IS**	-0.07 ± 0.97	-0.38 ± 1.39
**PASAT**	-1.17 ± 1.98	-1.47 ± 1.09

Abbreviations: RR-MS = relapsing-remitting MS; PP-MS = primary-progressive MS; CLVT = California Verbal Learning Test; SDMT = Symbol Digit Modalities Test; DKEFS-I = Delis—Kaplan Executive Function System Inhibition; DKEFS-IS = Delis—Kaplan Executive Function System Inhibition Switching; PASAT = Paced Auditory Serial Addition Test.

* p < 0.05

### Structural MRI analysis

None of the controls showed abnormalities on conventional MRI scans. Comparisons between controls and patients in terms of mean normalized brain volume (NBV), normalized white matter volume (NWMV), normalized gray matter volume (NGMV), T1- and T2-WWlesion volumes, mean cortical thickness (CTh) of the 7 cortical areas (temporal lobe—medial aspect, temporal lobe—lateral aspect, frontal lobe, parietal lobe, occipital lobe, cingulate cortex and insula) and deep grey nuclei (thalamus, caudate, putamen, pallidum), hippocampus and cerebellar volumes, are reported in Table 3. MS patients had significantly lower NBV (p = 0.01) and NGMV (p<0.001) but not NWMV. In regards to cortical thickness, MS patients had significantly lower thickness than controls in the temporal lobe—lateral aspect (p = 0.049), frontal lobe (p = 0.006), parietal lobe (p = 0.020), occipital lobe (p = 0.038), cingulate cortex (p = 0.006) and insula (p = 0.01). In terms of deep grey nuclei volume, MS patients had a significantly volume decrease in the thalamus (p = 0.005), caudate (p = 0.037), putamen (p = 0.021) and pallidum (p = 0.007).

**Table 3:** Mean and standard deviation of MRI measures for each subject group.

	Controls (n = 37)	All patients (n = 57)	RR-MS (n = 42)	PP-MS (n = 15)
**T2LV**	_	5.9 ± 8.7	4.5 ± 6.0	9.6 ± 13.3
**T1LV**	_	2.0 ± 4.1	1.2 ± 1.8	4.1 ± 7.1
**NBV**	1558.5 ± 148.8	1468.5 ± 137.6**	1489.6 ± 131.4	1406.6 ± 135.9
**NGMV**	843.2 ± 98.7	775.4 ± 73.8***	791.4 ± 68.2	729.2 ± 69.0
**NWMV**	715.3 ± 71.9	693.0 ± 95.9	698.3 ± 100.2	677.5 ± 79.7
**Temporal-MA**	3.2 ± 0.2	3.0 ± 0.3	3.1 ± 0.3	2.9 ± 0. **
**Temporal-LA**	2.8 ± 0.1	2.7 ± 0.2*	2.7 ± 0.2	2.6 ± 0. **
**Frontal lobe**	2.7 ± 0.1	2.6 ± 0.1**	2.6 ± 0.1*	2.5 ± 0. *
**Parietal lobe**	2.4 ± 0.1	2.3 ± 0.1*	2.4 ± 0.1	2.3 ± 0.2**
**Occipital lobe**	2.0 ± 0.1	1.9 ± 0.1*	1.9 ± 0.1	1.9 ± 0.1*
**cingulate**	2.7 ± 0.1	2.6 ± 0.1**	2.6 ± 0.1**	2.6 ± 0.2
**insula**	3.1 ± 0.1	3.0 ± 0.2**	3.0 ± 0.1**	3.0 ± 0.2*
**Cerebellum**	51.7 ± 5.8	50.0 ± 7.3	50.7 ± 7.4	47.8 ± 6.6*
**Thalamus**	7.2 ± 1.0	6.6 ± 1.0**	6.7 ± 1.1*	6.3 ± 0.8**
**Caudate**	3.5 ± 0.5	3.4 ± 0.5*	3.4 ± 0.5	3.1 ± 0.4 ** #
**Putamen**	5.1 ± 0.8	4.7 ± 1.0	4.9 ± 1.1	4.1 ± 0.5***
**Pallidum**	1.6 ± 0.2	1.4 ± 0.3	1.4 ± 0.3*	1.4 ± 0.1**
**Hippocampus**	4.2 ± 0.4	4.0 ± 0.5	4.1 ± 0.5	3.9 ± 0.5*
**Amygdala**	1.6 ± 0.2	1.5 ± 0.3	1.6 ± 0.3	1.4 ± 0.2**

RR-MS = relapsing-remitting MS; PP-MS = primary-progressive MS; All patients = both patients groups together (RR-MS & PP-MS); T2LV = T2-weighted lesion volume; T1LV = T1-weighted lesion volume; NBV = normalized brain volume; NGMV = normalized gray matter volume; NWMV = normalized white matter volume; Temporal MA = temporal lobe -medial aspect; Temporal LA = Temporal lobe—lateral aspect. Normalized volumes are in mL, cortical thickness is in mm, subcortical volume is in mm^3^.

* p < 0.05 when compared with control group

** p < 0.01 when compared with control group

*** p < 0.001 when compared with control group

^#^ p < 0.05 when compared with RR-MS group

Between patient disease types (RR-MS vs PP-MS), when correcting for age, there were no significant differences between PP-MS patients and RR-MS patients with regard to NBV, NGMV, NWMV, T1- and T2-W lesion volumes. Although PP-MS patients had less cortical thickness than RR-MS patients in all seven regions, none of these reached the required level of significance. In regards to deep GM volumes, PP-MS patients had greater volume reductions in nearly all areas (cerebellum, thalamus, caudate, putamen, hippocampus and amygdala) compared to RR-MS patients, only the difference in caudate volume reached a level of significance (p = 0.041).

### Correlations of MRI measurements and neuropsychological tests

After correcting for age, gender and disease duration, the RR-MS patient group showed significant correlations between overall cognitive performance and NBV (r = 0.37, p = 0.022), NGMV (r = 0.38, p = 0.017) and temporal lobe—lateral aspect (r = 0.33, p = 0.040). The SDMT correlated significantly with NBV (r = 0.34, p = 0.033), NGMV (r = 0.38, p = 0.016), temporal lobe—medial aspect (r = 0.34, p = 0.035), temporal lobe—lateral aspect (r = 0.38, p = 0.017) and parietal lobe (r = 0.47, p = 0.002). D-KEFS Inhibition correlated significantly with T1LV (r = -0.41, p = 0.010), T2LV (r = -0.37, p = 0.020), temporal lobe—lateral aspect (r = 0.34, p = 0.034) and frontal lobe (r = 0.44, p = 0.005). D-KEFS Inhibition Switching correlated significantly with NBV (r = 0.36, p = 0.023) and NWMV (r = 0.32, p = 0.044).

The PP-MS patient group showed significant correlations between overall cognitive performance and NBV (r = 0.60, p = 0.040), NWMV (r = 0.73, p = 0.008), thalamus (r = 0.72, p = 0.012) and putamen (r = 0.66, p = 0.027). The SDMT correlated significantly with NBV (r = 0.70, p = 0.011), NWMV (r = 0.75, p = 0.005), T1LV (r = -0.58, p = 0.048), T2LV (r = -0.67, p = 0.017), thalamus (r = 0.74, p = 0.009) and putamen (r = 0.83, p = 0.001). D-KEFS Inhibition correlated significantly with thalamus (r = 0.70, p = 0.017) and hippocampus (r = 0.82, p = 0.002). D-KEFS Inhibition Switching correlated significantly with thalamus (r = 0.65, p = 0.029). The PASAT correlated significantly with volume of the putamen (r = 0.64, p = 0.33).

## Discussion

Our study aimed at exploring potential differences in cognitive performance and MRI correlates of cognitive deficits in patients with RR-MS and patients with PP-MS who are characterized by predominant features of inflammation and neurodegeneration, respectively. Clinically, our PP-MS patients were older, had longer disease duration and were more physically disabled than RR-MS patients [5,35]. With regard to cognitive performance, PP-MS patients showed worse scores than RR-MS patients on five out of six NP tests, although a significant difference was only found for verbal learning and memory. These findings are in agreement with those reported by other studies comparing NP performance between RR-MS and PP-MS, with PP-MS patients experiencing more severe and more widespread impairment than RR-MS patients [4,5,36]. Furthermore, in line with our results, a previous study by Gaudino et al. found that PP-MS patients had more difficulty with verbal learning than RR-MS patients [6].

With regard to MRI metrics, the MS patient group as a whole differed from the control group in terms of decreased NBV, NGMV and NWMV, although only the first two reached a level of significance in line with findings of previous studies [17,37–39]. Likewise, MS patients showed a significantly decreased volume of subcortical GM nuclei with a preferential pattern of atrophy in the thalamus, caudate, putamen and pallidum compared to healthy controls. These findings are in line with those of previous studies [40–44] and support the relevance of cortical and subcortical gray matter atrophy in MS [45]. In line with previous studies [9,10,46], the extent of GM involvement in MS patients was further supported by the presence of significant cortical thinning in almost all the examined cortical areas compared to controls. Although some studies found a more preferential pattern for the frontal and temporal lobes cortical thinning [47–49], other studies found a frontotemporal preferential pattern in cognitively normal MS patients, but a more widespread pattern in mild (and severe) cognitively impaired patients [17,50], which seems to better reflect our MS patient population.

When the two groups of patients were compared to each other, PP-MS patients showed higher T1- and T2-LV, which could be explained by the fact that the RR-MS patients enrolled in our study were in the early stage of the disease. Furthermore, PP-MS patients showed lower brain volume (NBV, NGMV, NWMV and deep GM structures) and cortical thickness compared to RR-MS patients, supporting the knowledge that cortical demyelination is predominant in the progressive stages of MS with longer disease duration [9,47,50]. However, only the difference in caudate volume reached statistical significance between the two groups. Previous studies have found caudate atrophy in MS compared to healthy controls [51–53] and one study did find caudate volume reduction in SPMS and PP-MS, but not in RR-MS patients [53]. The presence of significant atrophy of the deep gray matter nuclei in patients with progressive MS agrees with pathologic [40] and MR imaging results [54–56], showing neuronal loss of up to 30–35% in these structures.

The most interesting question addressed by our study is, perhaps, the possible differences in MRI correlates of cognitive impairment in patients with predominant inflammation and patients with predominant neurodegeneration. We found that cognitive performance in RR-MS was associated with NBV, NGMV, NWMV, lesion volume and cortical thickness of the temporal lobe, parietal lobe and frontal lobe while cognitive performance in PP-MS was associated with NBV, NWMV, lesion volume and the volumes of the thalamus, hippocampus and putamen. This suggests that, at least in our cohort, the presence of WM lesions as well as GM injury contributed to the cognitive status with MRI measures of cortical GM damage being the dominant correlate in RR-MS and MRI measures of subcortical GM injury in PP-MS. Studies investigating the relationship between cortical thickness and subcortical volumes with cognitive deficits are still sparse. A study with RR-MS patients found correlations between left hemispheric superior temporal gyrus thickness and general cognitive status, motor skills, attention, and information processing speed [48]. Another study with RR-MS patients found correlations between cognitive status (both mild and severe) and thinning of almost all cortical areas analyzed and more specific correlations between NP tests and the frontal and temporal lobe [50]. However, these studies did not include subcortical areas or PP-MS patients for comparison. Nevertheless, correlations between cognitive tests and subcortical GM volumes have been found in previous studies including both RR-MS and SPMS patients [42,44,57,58]. Cognitive impairment in MS is complex and results from several overlapping factors such as premorbid cognitive function, WM lesion volumes and worsening of tissue damage within lesions, lesional and non-lesional cortical and subcortical gray matter damage and adaptive or maladaptive functional reorganization. These factors are likely to play a different role depending on the stage of the disease and on the individual genetic background with WM lesion volume, location, and WM microscopic injury being predominant in the early relapsing stage of the disease while cortical and subcortical gray matter volume loss and gray matter lesions becoming predominant as the disease progresses toward secondary degeneration. The association between NP scores and thalamic volume decrease in our PP-MS group in absence of significant association with MRI measures of cortical degeneration is somehow unexpected. Since both cortical and deep GM degeneration are relevant in PP-MS [40,59], the fact that we found only a trend towards a significant correlation between measures of cognitive performance (DKEFS-IS) and measures of cortical frontal lobe thickness may be due to the small sample size.

Our study has a few limitations. Since the number of PP-MS patients was relatively small, our findings need to be interpreted with caution until studies with larger sample sizes become available. The number of CI patients seems rather meager (25%), however, this may be due to the relative short disease duration of our patient group (5.8 years for RR-MS patients and 6.8 years for the whole MS patient group). A previous study found a relationship between cognitive impairment and disease duration; after five years of disease duration 20.9% of patients scored 1SD below cut-off for impairment, after 10 years this increased to 29.3% [60]. Our study also used a more stringent cut-off at 1.65 SD. Furthermore, due to the retrospective nature of our study, we were unable to include NP tests covering visuospatial memory, which is often impaired in MS patients [61,62]. Nevertheless, we feel the current NP test battery reflects patients’ cognitive performance adequately. At the time of our patients’ enrollment, MRI sequences for GM lesion detection were not available on our scanner and, therefore, we could not assess the impact of cortical lesions on cognitive deficits. Previous studies have shown that cortical lesions represent a relevant correlate of both physical and cognitive disability [14,15,63]. However, WM lesions also play a relevant role in the development of cognitive deficits, possibly more relevant than previously assumed [64] Furthermore, the inclusion of MRI measures of microstructural tissue damage such as diffusion tensor imaging could provide a better interpretation of selective cognitive deficits, as suggested in patients with different clinical phenotypes [26,65]. Future research should involve larger cohorts of patients with progressive MS, and investigate in a longitudinal fashion whether the development of cognitive impairment in different groups of patients is associated with specific patterns of MRI metrics. This would help identify patients at higher risk of cognitive decline and allow early and tailored intervention with both pharmacological and rehabilitative treatments.

## References

[1] Compston A, Coles A. Multiple sclerosis. Lancet. 2008;372: 1502–17. 10.1016/S0140-6736(08)61620-7 18970977

[2] Noseworthy JH, Lucchinetti C, Rodriguez M, Weinshenker BG. Multiple sclerosis. N Engl J Med. 2000;343: 938–52. 10.1056/NEJM200009283431307 11006371

[3] Rao SM, Leo GJ, Ellington L, Nauertz T, Bernardin L, Unverzagt F. Cognitive dysfunction in multiple sclerosis. II. Impact on employment and social functioning. Neurology. 1991;41: 692–6. 1823781 10.1212/wnl.41.5.692

[4] Huijbregts SCJ, Kalkers NF, de Sonneville LMJ, de Groot V, Reuling IEW, Polman CH. Differences in cognitive impairment of relapsing remitting, secondary, and primary progressive MS. Neurology. 2004;63: 335–9. 15277630 10.1212/01.wnl.0000129828.03714.90

[5] Ruet A, Deloire M, Charré-Morin J, Hamel D, Brochet B. Cognitive impairment differs between primary progressive and relapsing-remitting MS. Neurology. 2013;80: 1501–8. 10.1212/WNL.0b013e31828cf82f 23516324

[6] Gaudino EA, Chiaravalloti ND, DeLuca J, Diamond BJ. A comparison of memory performance in relapsing-remitting, primary progressive and secondary progressive, multiple sclerosis. Neuropsychiatry Neuropsychol Behav Neurol. 2001;14: 32–44. 11234907

[7] Potagas C, Giogkaraki E, Koutsis G, Mandellos D, Tsirempolou E, Sfagos C, et al Cognitive impairment in different MS subtypes and clinically isolated syndromes. J Neurol Sci. 2008;267: 100–6. 10.1016/j.jns.2007.10.002 17997417

[8] Kuchling J, Ramien C, Bozin I, Dörr J, Harms L, Rosche B, et al Identical lesion morphology in primary progressive and relapsing-remitting MS -an ultrahigh field MRI study. Mult Scler. 2014;20: 1866–71. 10.1177/1352458514531084 24781284

[9] Kutzelnigg A, Lucchinetti CF, Stadelmann C, Brück W, Rauschka H, Bergmann M, et al Cortical demyelination and diffuse white matter injury in multiple sclerosis. Brain. 2005;128: 2705–12. 10.1093/brain/awh641 16230320

[10] Ceccarelli A, Rocca MA, Pagani E, Colombo B, Martinelli V, Comi G, et al A voxel-based morphometry study of grey matter loss in MS patients with different clinical phenotypes. Neuroimage. 2008;42: 315–22. 10.1016/j.neuroimage.2008.04.173 18501636

[11] Amato MP, Bartolozzi ML, Zipoli V, Portaccio E, Mortilla M, Guidi L, et al Neocortical volume decrease in relapsing-remitting MS patients with mild cognitive impairment. Neurology. 2004;63: 89–93. 15249616 10.1212/01.wnl.0000129544.79539.d5

[12] Camp SJ, Stevenson VL, Thompson AJ, Miller DH, Borras C, Auriacombe S, et al Cognitive function in primary progressive and transitional progressive multiple sclerosis: a controlled study with MRI correlates. Brain. 1999;122 (Pt 7: 1341–8. 10388799 10.1093/brain/122.7.1341

[13] Benedict RHB, Weinstock-Guttman B, Fishman I, Sharma J, Tjoa CW, Bakshi R. Prediction of neuropsychological impairment in multiple sclerosis: comparison of conventional magnetic resonance imaging measures of atrophy and lesion burden. Arch Neurol. 2004;61: 226–30. 10.1001/archneur.61.2.226 14967771

[14] Calabrese M, Agosta F, Rinaldi F, Mattisi I, Grossi P, Favaretto A, et al Cortical lesions and atrophy associated with cognitive impairment in relapsing-remitting multiple sclerosis. Arch Neurol. 2009;66: 1144–50. 10.1001/archneurol.2009.174 19752305

[15] Calabrese M, Poretto V, Favaretto A, Alessio S, Bernardi V, Romualdi C, et al Cortical lesion load associates with progression of disability in multiple sclerosis. Brain. 2012;135: 2952–61. 10.1093/brain/aws246 23065788

[16] Calabrese M, Favaretto A, Martini V, Gallo P. Grey matter lesions in MS: from histology to clinical implications. Prion. 2012;7: 20–7. 10.4161/pri.22580 23093801 PMC3609046

[17] Morgen K, Sammer G, Courtney SM, Wolters T, Melchior H, Blecker CR, et al Evidence for a direct association between cortical atrophy and cognitive impairment in relapsing-remitting MS. Neuroimage. 2006;30: 891–8. 10.1016/j.neuroimage.2005.10.032 16360321

[18] Brück W, Lucchinetti C, Lassmann H. The pathology of primary progressive multiple sclerosis. Mult Scler. 2002;8: 93–7. 11990878 10.1191/1352458502ms785rr

[19] Polman CH, Reingold SC, Edan G, Filippi M, Hartung H-P, Kappos L, et al Diagnostic criteria for multiple sclerosis: 2005 revisions to the “McDonald Criteria”. Ann Neurol. 2005;58: 840–6. 10.1002/ana.20703 16283615

[20] Kurtzke JF. Rating neurologic impairment in multiple sclerosis: an expanded disability status scale (EDSS). Neurology. 1983;33: 1444–52. 6685237 10.1212/wnl.33.11.1444

[21] Spreen O. & Benton AL. Neurosensory Center Comprehensive Examination for Aphasia: Manual of instructions (NCCEA). rev. ed Victoria BC: University of Victoria; 1977.

[22] Delis D.C., Kramer J.H., Kaplan E., & Ober BA. The California Verbal Learning Test. San Antonio, TX: Psychological Corporation; 1987.

[23] Smith A. Symbol Digits Modalities Test. Los Angeles, CA: Western Psychological Services; 1982.

[24] Gronwall DM. Paced auditory serial-addition task: a measure of recovery from concussion. Percept Mot Skills. 1977;44: 367–73. 10.2466/pms.1977.44.2.367 866038

[25] Delis D. C., Kaplan E. and Kramer J. Delis Kaplan Executive Function System. San Antonio, TX: The Psychological Corporation; 2001.

[26] Bester M, Lazar M, Petracca M, Babb JS, Herbert J, Grossman RI, et al Tract-specific white matter correlates of fatigue and cognitive impairment in benign multiple sclerosis. J Neurol Sci. 2013;330: 61–6. 10.1016/j.jns.2013.04.005 23643443 PMC4651179

[27] Derogatis LR, Melisaratos N. The Brief Symptom Inventory: an introductory report. Psychol Med. 1983;13: 595–605. 6622612

[28] Smets EM, Garssen B, Bonke B, De Haes JC. The Multidimensional Fatigue Inventory (MFI) psychometric qualities of an instrument to assess fatigue. J Psychosom Res. 1995;39: 315–25. 7636775 10.1016/0022-3999(94)00125-o

[29] Van Waesberghe JH, van Walderveen MA, Castelijns JA, Scheltens P, Lycklama à Nijeholt GJ, Polman CH, et al Patterns of lesion development in multiple sclerosis: longitudinal observations with T1-weighted spin-echo and magnetization transfer MR. AJNR Am J Neuroradiol. 1998;19: 675–83. 9576653 PMC8337386

[30] De Stefano N, Matthews PM, Filippi M, Agosta F, De Luca M, Bartolozzi ML, et al Evidence of early cortical atrophy in MS: relevance to white matter changes and disability. Neurology. 2003;60: 1157–62. 12682324 10.1212/01.wnl.0000055926.69643.03

[31] Smith KJ, Lassmann H. The role of nitric oxide in multiple sclerosis. Lancet Neurol. 2002;1: 232–41. 12849456 10.1016/s1474-4422(02)00102-3

[32] Battaglini M, Jenkinson M, De Stefano N. Evaluating and reducing the impact of white matter lesions on brain volume measurements. Hum Brain Mapp. 2012;33: 2062–71. 10.1002/hbm.21344 21882300 PMC6870255

[33] Fischl B, Sereno MI, Dale AM. Cortical surface-based analysis. II: Inflation, flattening, and a surface-based coordinate system. Neuroimage. 1999;9: 195–207. 10.1006/nimg.1998.0396 9931269

[34] Desikan RS, Ségonne F, Fischl B, Quinn BT, Dickerson BC, Blacker D, et al An automated labeling system for subdividing the human cerebral cortex on MRI scans into gyral based regions of interest. Neuroimage. 2006;31: 968–80. 10.1016/j.neuroimage.2006.01.021 16530430

[35] Feys P, Bibby BM, Baert I, Dalgas U. Walking capacity and ability are more impaired in progressive compared to relapsing type of multiple sclerosis. Eur J Phys Rehabil Med. 2014; 25180640

[36] De Sonneville LMJ, Boringa JB, Reuling IEW, Lazeron RHC, Adèr HJ, Polman CH. Information processing characteristics in subtypes of multiple sclerosis. Neuropsychologia. 2002;40: 1751–65. 12062887 10.1016/s0028-3932(02)00041-6

[37] Tiberio M, Chard DT, Altmann DR, Davies G, Griffin CM, Rashid W, et al Gray and white matter volume changes in early RRMS: a 2-year longitudinal study. Neurology. 2005;64: 1001–7. 10.1212/01.WNL.0000154526.22878.30 15781816

[38] Carone DA, Benedict RHB, Dwyer MG, Cookfair DL, Srinivasaraghavan B, Tjoa CW, et al Semi-automatic brain region extraction (SABRE) reveals superior cortical and deep gray matter atrophy in MS. Neuroimage. 2006;29: 505–14. 10.1016/j.neuroimage.2005.07.053 16169253

[39] Calabrese M, Battaglini M, Giorgio A, Atzori M, Bernardi V, Mattisi I, et al Imaging distribution and frequency of cortical lesions in patients with multiple sclerosis. Neurology. 2010;75: 1234–40. 10.1212/WNL.0b013e3181f5d4da 20739644

[40] Cifelli A, Arridge M, Jezzard P, Esiri MM, Palace J, Matthews PM. Thalamic neurodegeneration in multiple sclerosis. Ann Neurol. 2002;52: 650–3. 10.1002/ana.10326 12402265

[41] Sepulcre J, Sastre-Garriga J, Cercignani M, Ingle GT, Miller DH, Thompson AJ. Regional gray matter atrophy in early primary progressive multiple sclerosis: a voxel-based morphometry study. Arch Neurol. 2006;63: 1175–80. 10.1001/archneur.63.8.1175 16908748

[42] Houtchens MK, Benedict RHB, Killiany R, Sharma J, Jaisani Z, Singh B, et al Thalamic atrophy and cognition in multiple sclerosis. Neurology. 2007;69: 1213–23. 10.1212/01.wnl.0000276992.17011.b5 17875909

[43] Ramasamy DP, Benedict RHB, Cox JL, Fritz D, Abdelrahman N, Hussein S, et al Extent of cerebellum, subcortical and cortical atrophy in patients with MS: a case-control study. J Neurol Sci. 2009;282: 47–54. 10.1016/j.jns.2008.12.034 19201003

[44] Batista S, Zivadinov R, Hoogs M, Bergsland N, Heininen-Brown M, Dwyer MG, et al Basal ganglia, thalamus and neocortical atrophy predicting slowed cognitive processing in multiple sclerosis. J Neurol. 2012;259: 139–46. 10.1007/s00415-011-6147-1 21720932

[45] Inglese M, Oesingmann N, Casaccia P, Fleysher L. Progressive multiple sclerosis and gray matter pathology: an MRI perspective. Mt Sinai J Med. 78: 258–67. 10.1002/msj.20247 21425269 PMC3079372

[46] Liu Y, Xie T, He Y, Duan Y, Huang J, Ren Z, et al Cortical thinning correlates with cognitive change in multiple sclerosis but not in neuromyelitis optica. Eur Radiol. 2014;24: 2334–43. 10.1007/s00330-014-3239-1 24906701

[47] Sailer M, Fischl B, Salat D, Tempelmann C, Schönfeld MA, Busa E, et al Focal thinning of the cerebral cortex in multiple sclerosis. Brain. 2003;126: 1734–44. 10.1093/brain/awg175 12805100

[48] Achiron A, Chapman J, Tal S, Bercovich E, Gil H, Achiron A. Superior temporal gyrus thickness correlates with cognitive performance in multiple sclerosis. Brain Struct Funct. 2013;218: 943–50. 10.1007/s00429-012-0440-3 22790785

[49] Narayana PA, Govindarajan KA, Goel P, Datta S, Lincoln JA, Cofield SS, et al Regional cortical thickness in relapsing remitting multiple sclerosis: A multi-center study. NeuroImage Clin. 2012;2: 120–31. 10.1016/j.nicl.2012.11.009 24179765 PMC3777814

[50] Calabrese M, Rinaldi F, Mattisi I, Grossi P, Favaretto A, Atzori M, et al Widespread cortical thinning characterizes patients with MS with mild cognitive impairment. Neurology. 2010;74: 321–8. 10.1212/WNL.0b013e3181cbcd03 20101038

[51] Bermel RA, Innus MD, Tjoa CW, Bakshi R. Selective caudate atrophy in multiple sclerosis: a 3D MRI parcellation study. Neuroreport. 2003;14: 335–9. 10.1097/01.wnr.0000059773.23122.ce 12634479

[52] Prinster A, Quarantelli M, Orefice G, Lanzillo R, Brunetti A, Mollica C, et al Grey matter loss in relapsing-remitting multiple sclerosis: a voxel-based morphometry study. Neuroimage. 2006;29: 859–67. 10.1016/j.neuroimage.2005.08.034 16203159

[53] Pagani E, Rocca MA, Gallo A, Rovaris M, Martinelli V, Comi G, et al Regional brain atrophy evolves differently in patients with multiple sclerosis according to clinical phenotype. AJNR Am J Neuroradiol. 2005;26: 341–6. 15709132 PMC7974082

[54] Bakshi R, Dmochowski J, Shaikh ZA, Jacobs L. Gray matter T2 hypointensity is related to plaques and atrophy in the brains of multiple sclerosis patients. J Neurol Sci. 2001;185: 19–26. 11266686 10.1016/s0022-510x(01)00477-4

[55] Inglese M, Liu S, Babb JS, Mannon LJ, Grossman RI, Gonen O. Three-dimensional proton spectroscopy of deep gray matter nuclei in relapsing-remitting MS. Neurology. 2004;63: 170–2. 15249633 10.1212/01.wnl.0000133133.77952.7c

[56] Rovaris M, Bozzali M, Iannucci G, Ghezzi A, Caputo D, Montanari E, et al Assessment of normal-appearing white and gray matter in patients with primary progressive multiple sclerosis: a diffusion-tensor magnetic resonance imaging study. Arch Neurol. 2002;59: 1406–12. 12223026 10.1001/archneur.59.9.1406

[57] Benedict RHB, Ramasamy D, Munschauer F, Weinstock-Guttman B, Zivadinov R. Memory impairment in multiple sclerosis: correlation with deep grey matter and mesial temporal atrophy. J Neurol Neurosurg Psychiatry. 2009;80: 201–6. 10.1136/jnnp.2008.148403 18829629

[58] Modica CM, Zivadinov R, Dwyer MG, Bergsland N, Weeks AR, Benedict RHB. Iron and volume in the deep gray matter: association with cognitive impairment in multiple sclerosis. AJNR Am J Neuroradiol. 2015;36: 57–62. 10.3174/ajnr.A3998 24948507 PMC7965904

[59] Inglese M, Park S-J, Johnson G, Babb JS, Miles L, Jaggi H, et al Deep gray matter perfusion in multiple sclerosis: dynamic susceptibility contrast perfusion magnetic resonance imaging at 3 T. Arch Neurol. 2007;64: 196–202. 10.1001/archneur.64.2.196 17296835

[60] Achiron A, Chapman J, Magalashvili D, Dolev M, Lavie M, Bercovich E, et al Modeling of cognitive impairment by disease duration in multiple sclerosis: a cross-sectional study. PLoS One. 2013;8: e71058 10.1371/journal.pone.0071058 23936485 PMC3731335

[61] Foong J, Rozewicz L, Quaghebeur G, Davie CA, Kartsounis LD, Thompson AJ, et al Executive function in multiple sclerosis. The role of frontal lobe pathology. Brain. 1997;120 (Pt 1: 15–26. 9055794 10.1093/brain/120.1.15

[62] Huijbregts SCJ, Kalkers NF, de Sonneville LMJ, de Groot V, Polman CH. Cognitive impairment and decline in different MS subtypes. J Neurol Sci. 2006;245: 187–94. 10.1016/j.jns.2005.07.018 16643951

[63] Calabrese M, De Stefano N, Atzori M, Bernardi V, Mattisi I, Barachino L, et al Detection of cortical inflammatory lesions by double inversion recovery magnetic resonance imaging in patients with multiple sclerosis. Arch Neurol. 2007;64: 1416–22. 10.1001/archneur.64.10.1416 17923625

[64] Papadopoulou A, Müller-Lenke N, Naegelin Y, Kalt G, Bendfeldt K, Kuster P, et al Contribution of cortical and white matter lesions to cognitive impairment in multiple sclerosis. Mult Scler. 2013;19: 1290–6. 10.1177/1352458513475490 23459568

[65] Hulst HE, Steenwijk MD, Versteeg A, Pouwels PJW, Vrenken H, Uitdehaag BMJ, et al Cognitive impairment in MS: impact of white matter integrity, gray matter volume, and lesions. Neurology. 2013;80: 1025–32. 10.1212/WNL.0b013e31828726cc 23468546

